# Catabolic pathways regulated by mTORC1 are pivotal for survival and growth of cancer cells expressing mutant Ras

**DOI:** 10.18632/oncotarget.6334

**Published:** 2015-11-14

**Authors:** Suhyun Sung, Jungwon Choi, Heesun Cheong

**Affiliations:** ^1^ Comparative Biomedicine Research Branch, Division of Cancer Biology, National Cancer Center, Ilsandong-gu, Goyang-si, Gyeonggi-do, Republic of Korea

**Keywords:** autophagy, macropinocytosis, mTORC1, KRas, pancreatic ductal adenocarcinoma (PDA)

## Abstract

Oncogenic Ras stimulates macropinocytosis, a clathrin-independent endocytosis that increases the uptake of extracellular fluid. However, the functional significance of and regulatory mechanisms driving macropinocytosis in cancer cells remain largely unknown. Here, we show that extracellular macromolecules, such as albumin, internalized by Ras-expressing cells can support growth and survival under the nutrient-deprived conditions like those found in tumors. Moreover, we demonstrate that autophagy, a lysosome-mediated catabolic pathway, is required for the uptake and degradation of macropinocytic vesicles. Intracellular metabolites derived from macropinocytosis and autophagy directly influence the activity and localization of mTOR, which is ultimately responsible for the restoration of cell growth. Surprisingly, suppression of mTORC1, which typically triggers anabolic processes, facilitates macropinocytosis and thus supports cell growth and survival under the nutrient-deprived conditions. In a mouse xenograft model of pancreatic ductal adenocarcinoma, concomitant inhibition of macropinocytosis/autophagy and mTOR activity resulted in antitumor effects. These data suggest that novel anti-cancer strategies interrupting these metabolic processes and related signaling molecules may represent promising therapeutic avenues.

## INTRODUCTION

Macropinocytosis is a clathrin-independent endocytic pathway that internalizes macromolecule-rich extracellular fluid without the need for specific vesicle-coat proteins. A variety of growth factor signals induce macropinocytosis, which facilitates the protrusion of plasma membrane ruffles and allows the internalization of engulfed extracellular fluid. This process is most well defined in dendritic cells and macrophages involved in MHC antigen presentation. Macropinocytosis also contributes to cancer cell growth and survival by maintaining nutrient balance [1, 2]. Oncogenic Ras expression induces macropinocytosis and supports cellular demand for metabolites, such as amino acids, during metabolic stress that results from the rapid growth of cancers [3].

Ras activation also promotes cancer cell proliferation and survival and helps fulfill metabolic demand by inducing autophagy [4]. Autophagy is a pathway in which lysosomes degrade and recycle cytosolic components and organelles. This helps maintain intracellular homeostasis and serves as an adaptive survival mechanism in response to environmental changes. The role of autophagy in cancer is complex and dependent on tumor stage. For example, autophagy suppresses tumorigenesis during early cancer development, while in advanced tumors it promotes tumor progression [5, 6].

Autophagy is controlled by multiple growth-signaling molecules, including the serine/threonine protein kinase mTOR, whose activity promotes cell growth through multiple anabolic alterations. For example, mTOR activation suppresses autophagy under conditions that favor growth, while mTOR inactivation in response to nutrient or growth factor deprivation induces autophagy [7]. Notably, recent reports suggest that, despite the dominant anabolic features of the oncogenic Ras mutant, the conditions that activate oncogenic Ras also stimulate autophagy [4, 8]. This Ras-induced autophagy ameliorates the cellular metabolic stress that results from nutrient deprivation, suggesting that autophagy plays a key role in the metabolic regulation of oncogenic Ras-mediated cancer.

Oncogenic Ras increases the rate of glycolysis and stimulates the pentose phosphate pathway (PPP) and the hexosamine biosynthesis pathway (HBP), thereby promoting efficient macromolecule synthesis and rapid cellular proliferation [9]. Glutamine is also required for the growth of oncogenic Ras-mediated cancers such as pancreatic ductal adenocarcinoma (PDA) [10]. However, metabolic flux analysis utilizing carbon-labeled glucose and glutamine revealed an increase in levels of unlabeled metabolic intermediates for macromolecule synthesis in the presence of an oncogenic Ras mutant (unpublished observations). These data suggest that macromolecule catabolism is an important source of energy in cells with active oncogenic Ras. Proteins internalized through macropinocytosis could contribute to amino acid supplies in cancer cells, which could promote growth and survival in Ras-mediated cancer [3]. However, the regulation of Ras-mediated macropinocytosis in cancer is not fully understood.

Here, we examine the contributions of macropinocytosis and autophagy to catabolic processes that support growth and survival in cells expressing mutant Ras. Furthermore, we examine the importance of autophagy in the degradation of extracellular macromolecules taken up via macropinocytosis using autophagy defective Ras-expressing cells. We also determine whether the products of macropinocytosis and autophagy impact mTORC1 activity, and whether this activity in turn alters uptake and degradation of extracellular proteins. If molecules generated via macropinocytosis and autophagy help meet cellular metabolic needs and reactivate the growth signaling molecule mTORC1, then these processes may increase cell growth and survival in oncogenic Ras-driven cancer.

## RESULTS

### Oncogenic Ras expression increases macromolecule uptake via macropinocytosis

**Figure 1:**
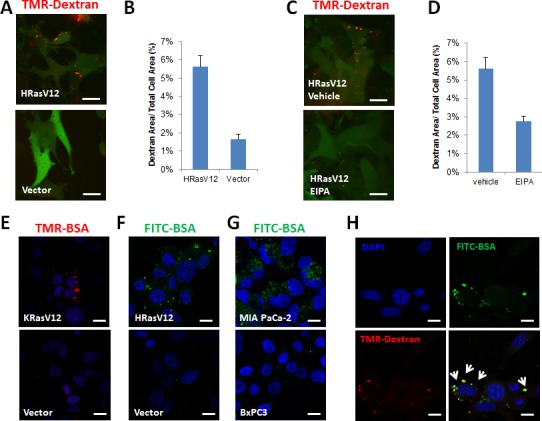
Oncogenic Ras-expression induces macropinocytosis and increases the uptake of extracellular proteins **A.** Macropinocytosis uptake assay utilizing TMR-dextran as a marker of macropinocytosis. Ectopic expression of the HRas G12V mutant in mouse embryonic fibroblast (MEF) cells is reported as intracellular GFP expression. Scale bars, 20 μm. **B.**The level of macropinocytic uptake is quantified by image-based determination of the total macropinocytic vesicle area compared with the whole cell area. Data are expressed as a percentage of the whole Ras-expressing cell area. **C.** Macropinocytic uptake of TMR-dextran in MEFs expressing HRas G12V is inhibited by 50 μM EIPA. **D.** Quantification of macropinocytic uptake in MEFs expressing HRas G12V treated with 50 μM EIPA. Data are expressed as a percentage of the whole cell area. **E.** Intracellular uptake of TMR-BSA in MEFs expressing KRas G12V **F.** FITC-BSA in MEFs expressing HRas G12V. **G.** Intracellular uptake of FITC-BSA in a PDA cell line, MIA PaCa-2, harboring K-Ras G12C compared with BxPC3 cells harboring wild type K-Ras. **H.** Internalized FITC-BSA in vesicles mostly co-localizes with TMR-dextran, as shown by the white arrow. Images shown are representative of at least 3 independent experiments.

Macropinocytosis increased the uptake of macromolecules induced by oncogenic Ras expression, as previously reported [3]. Fluorescent microscopy analysis of tetramethylrhodamine-conjugated high molecular weight dextran (TMR-dextran, 10KDa) uptake was used to examine the uptake rate of macropinocytic vesicles. MEFs harboring HRas G12V displayed increased macropinocytosis compared with normal MEF transfected with vector alone (Figure 1A, 1B). This phenotype was significantly inhibited by 5-N-ethyl-N-isopropyl-amiloride (EIPA), an amiloride analog known to inhibit Na^+^-H^+^ exchange in cells (Figure 1C, 1D). The internalization of dextran was quantified by comparison with the total area of cells expressing a GFP reporter or the DAPI stained area.

Macropinocytosis substantially elevated uptake of proteins such as bovine serum albumin (BSA) from the extracellular fluid, as visualized using fluorescent molecule-labeled BSA in MEFs harboring either KRas G12V (Figure 1E) or HRas G12V (Figure 1F). Cells from the MIA PaCa-2 pancreatic adenocarcinoma line, harboring a KRas mutant showed increased internalization of FITC-BSA. Conversely, BxPC3 cells expressing wild-type KRas did not show a significant increase in BSA uptake (Figure 1G). EIPA also inhibited BSA internalization in these cells. Consistent with previous data, internalized BSA was more likely to be co-localized with the macropinocytosis marker dextran (Figure 1H).

### Oncogenic Ras-induced macropinocytosis contributes to cell growth under nutrient deficiency

**Figure 2:**
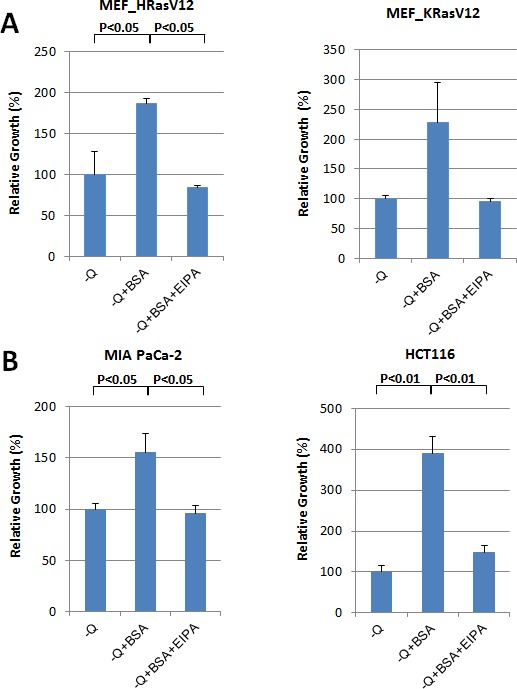

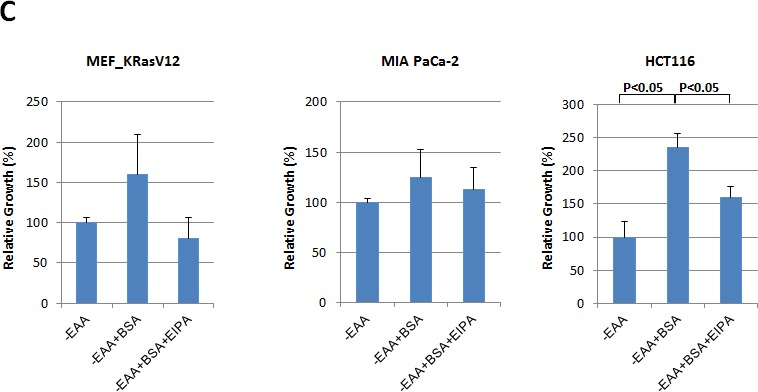
Oncogenic Ras-induced macropinocytosis contributes to cellular growth under nutrient deficiency **A.** In MEFs expressing KRas G12V or HRas G12V, the reduction in cell growth following glutamine deprivation is reversed by the treatment with 2% BSA. Cell growth after 6 days of glutamine (Q) deprivation with or without 2% BSA was quantified by cell counting. Furthermore, cell numbers after treatment with 25 μM EIPA to inhibit macropinocytic BSA uptake during conditions of starvation supplemented with 2% BSA were counted. Relative growth rate is presented after normalization by number of cells under each starvation condition without BSA. **B.** In the human cancer lines harboring mutant KRas (MIA PaCa-2 or HCT116), supplementing with 2% BSA reversed the reduction in growth resulting from glutamine deprivation. **C.** In response to essential amino acid (EAA) deprivation, growth of MEFs expressing KRas G12V, MiaPaCa2 and HCT116 cells was restored by treatment with 2% BSA. Data are presented relative to the values observed in the glutamine or EAA withdrawal conditions. Error bars indicate mean +/− SEM for *n* = 3 independent experiments. Statistical significance was determined via a student's *t*-test.

As shown in Figure 2, oncogenic Ras-induced macropinocytosis enhanced the uptake of extracellular BSA, rescued the cells from apoptosis and supported cell growth following either glutamine or essential amino acid (EAA) deprivation (Figure 2; Supplemental Figure 1). EIPA treatment inhibited this effect of BSA treatment in Ras-expressing cells (Figure 2; Supplemental Figure 1).

### Oncogenic Ras-induced macromolecule degradation requires autophagy

Although the treatment with Bafilomycin A1 blocked oncogenic Ras-mediated macropinocytosis in a previous report [3], it is not yet clear whether autophagy pathway plays a direct role in degradation of extracellular macromolecules subsequent to macropinocytosis.

**Figure 3:**
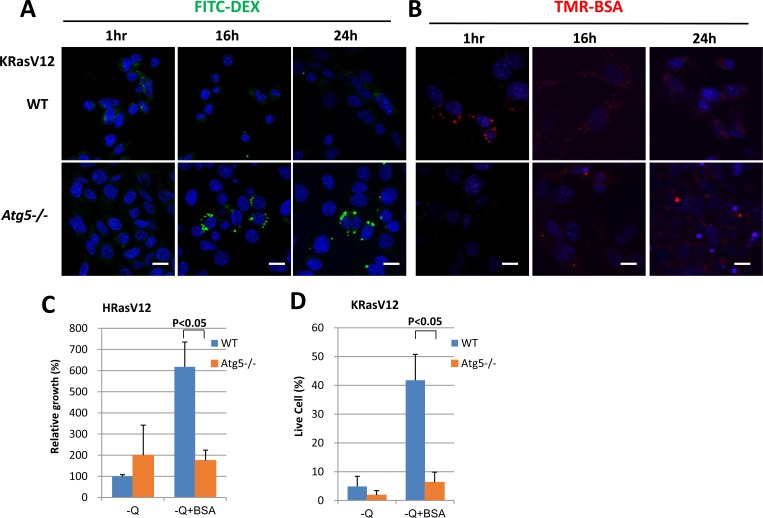
Oncogenic Ras-induced macromolecule degradation requires autophagy **A.** Uptake of FITC-Dextran in WT MEFs and *atg5* knockout (Atg5−/−) MEFs expressing KRas G12V was monitored at the indicated times. Scale bars, 20 μm. **B.** Uptake of TMR-BSA was also assessed in condition **A.**. Scale bars, 20 μm. **C.** HRas G12V-expressing WT MEFs and *atg5−/−*MEFs were each cultured in glutamine deprivation medium for 6 days, either with or without 2% BSA, and growth levels were compared by cell counting. Data are expressed relative to the values obtained for the glutamine deprivation conditions for each cell line. **D.** KRas G12V-expressing WT MEFs and *atg5−/−*MEFs were each cultured in glutamine deprivation medium for 6 days, either with or without 2% BSA, and live cell portion were measured by Annexin V and PI negative staining. Data are shown as the average of 3 independent experiments +/−SEM. Statistical significance was determined via a student's *t*-test.

Initially, internalization rates of FITC-Dextran (Figure 3A) and TMR-BSA (Figure 3B) were slightly inhibited in oncogenic Ras-expressing, autophagy deficient *atg5-*knockout (*Atg5−/−*) MEF cells. However, as incubation with 2% BSA continued over 12 h, FITC-Dextran and TMR-BSA in these cells accumulated within relatively larger vesicles rather than disappearing into macropinocytic vesicles as seen in MEFs with wild-type autophagy genes (Figure 3A, 3B). In addition, MIA PaCa-2 cells stably expressing Atg7 shRNA displayed increased accumulation of FITC-Dextran in vesicles after 16 h of incubation as compared to MIA PaCa-2 cells expressing control shRNA (Supplemental Figure 2). In contrast, ULK1/2 double knockout (DKO) autophagy deficient MEFs showed levels of FITC-Dextran accumulation comparable to WT autophagy MEFs under long-term starvation conditions (Supplemental Figure 3).

Next, we examined whether autophagy coupled with Ras expression plays a role in cell growth under amino acid starvation conditions supplemented with BSA. Compared with autophagy-intact cells, *atg5* KO cells with oncogenic Ras expression showed reduced growth in the glutamine deprived media, even in the presence of BSA (Figure 3C). Also apoptotic cell portion due to glutamine deprivation was restored by treatment with BSA in autophagy-intact cells but not in *atg5* KO cells (Figure 3D).

### mTORC1 inhibition increases macropinocytosis

**Figure 4:**
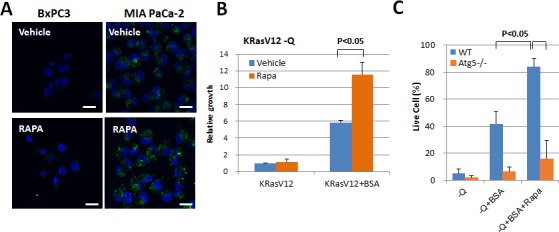
mTORC1 inhibition increases macropinocytosis **A.** The effect of rapamycin treatment on the uptake of FITC-BSA in MIA PaCa-2 (KRas mutant) cells. Scale bars, 20 μm **B.** In MEFs expressing KRas G12V cells, treatment with 2% BSA for 6 days reversed the decrease in growth that resulted from glutamine deprivation. Quantification of relative growth in MEFs expressing KRas G12V cells treated with 5 μM rapamycin is shown by cell counting. Data are presented relative to the values observed in glutamine-deprived conditions. **C.** KRas G12V-expressing WT MEFs and *atg5−/−*MEFs were each cultured in glutamine deprivation medium for 6 days, either with or without 2% BSA and with 2%BSA plus 5 μM rapamycin. Live cell portion was measured by Annexin V and PI negative staining. Error bars indicate mean +/− SEM for *n* = 3 independent experiments. Statistical significance was determined via a student's *t*-test.

We also examined the effect of autophagy activation on macropinocytosis, as autophagy induced by oncogenic Ras can be critical for tumor progression *in vivo* (Supplemental Figure 4). When rapamycin, an autophagy-activating mTOR inhibitor, was incubated with oncogenic Ras-expressing cells, FITC-BSA uptake increased significantly (Figure 4A). In addition to rapamycin treatment, the expression of Raptor, an essential component of the mTORC1 complex, was knocked down using shRNA in mutant HRas-expressing MEF cells. Macropinocytic vesicles harboring TMR-Dextran were substantially increased in MEFs expressing Raptor shRNA as compared to the cells with control shRNA (Supplemental Figure 5). Moreover, in cells whose growth was diminished by long-term deprivation of glutamine, growth and survival were restored after treatment with rapamycin (Figure 4B and Figure 4C).

### Oncogenic Ras-induced macromolecule degradation activates mTORC1

**Figure 5:**
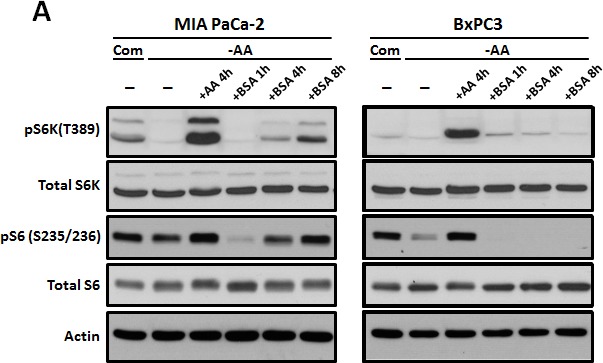

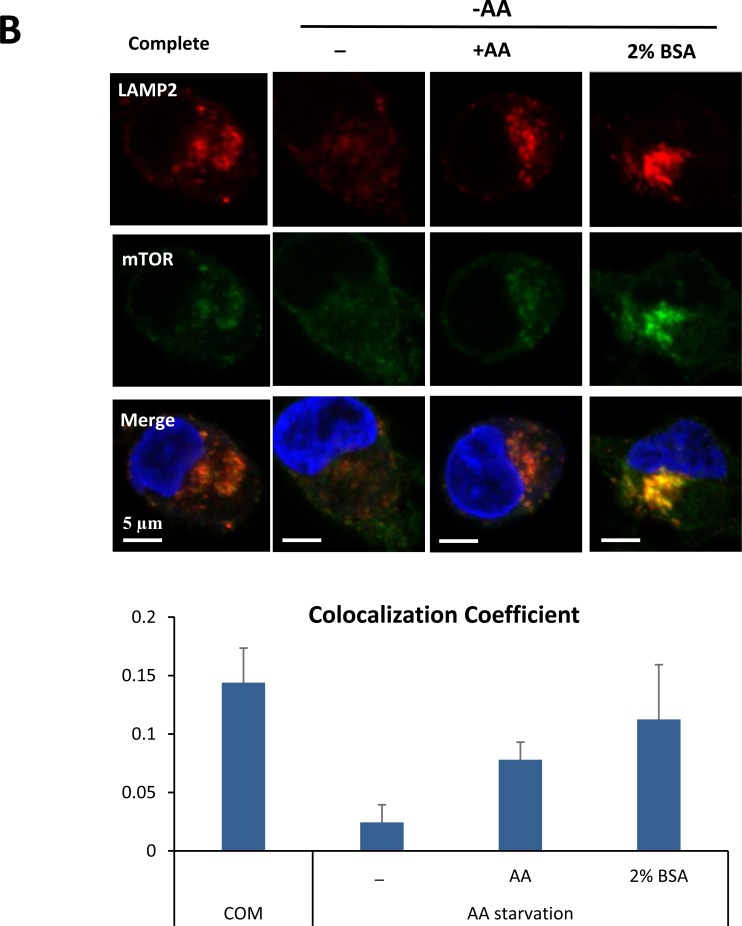

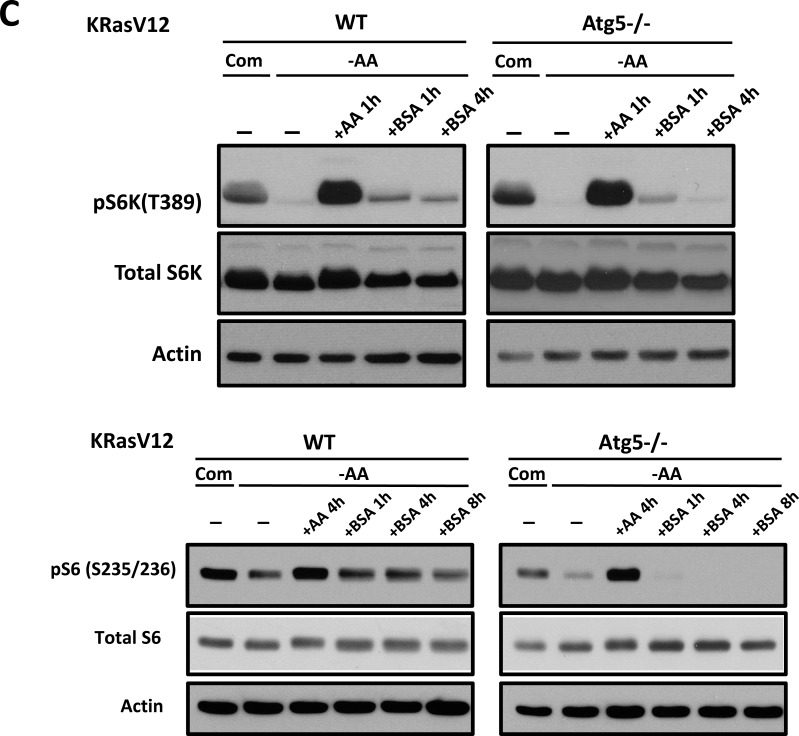
Oncogenic Ras-induced macromolecule degradation activates cellular nutrient sensing pathways **A.** MIA PaCa-2 harboring KRas G12C was cultured in amino acid-free medium for 1 hour and treated with total amino acids or 2% BSA for the indicated times. At the indicated times, mTOR activity was monitored by detection of phospho-p70S6 kinase (T389) and phospho-S6 (S235/236) using Western blotting. **B.** mTOR localization was monitored in at the indicated times, utilizing immune-fluorescence analysis. Supplementing the medium with 2% BSA restored mTOR localization to the normal state. Scale bars, 5 μm. **C.** KRasV12-expressing MEFs and *atg5* KO MEFs were deprived of amino acids and mTOR activity was monitored in the presence or absence of 2% BSA. Treatment with total amino acids was used as a positive control.

To investigate whether the uptake of extracellular proteins through macropinocytosis can influence mTORC1 activity, phosphorylation levels of the downstream targets p70 S6 kinase at T389 (S6K) and S6 ribosomal protein at S235/236 (S6) were monitored using Western blotting. mTOR activity is usually down-regulated under conditions of amino acid starvation, which is also well-known to activate autophagy. When BSA was added to the culture during amino acid withdrawal, S6K phosphorylation increased and was similar to that seen under nutrient-complete conditions. The addition of BSA during amino acid deprivation restored mTORC1 activity in MIA PaCa-2 cells with the KRas mutant allele, but not in BxPC3 harboring KRas wild type allele (Figure 5A).

According to recent reports, the subcellular localization of mTOR is altered depending on the intracellular nutrient status, particularly on the amino acid levels [11]. The majority of mTOR was distributed throughout the cytoplasm during amino acid deprivation; under nutrient rich conditions, the majority of mTOR was located in lysosomes, as indicated by co-localization with a lysosomal membrane protein, LAMP2. Four hours of BSA treatment resulted of the movement of mTOR protein into the lysosomes, even under amino acid starvation conditions (Figure 5B).

Finally, we analyzed whether autophagy influences the restoration of mTOR activity through degradation of internalized BSA. Both WT and *atg5* KO MEFs harboring oncogenic KRas were cultured in the media devoid of amino acids and then incubated further after the addition of 2% BSA. The reduction in phosphorylation of p70 S6 kinase (S6K) and S6 ribosomal protein (S6) due to amino acid starvation was reversed in WT MEFs at the indicated time points following the addition of BSA. However, phospho-S6K and -S6 levels in *atg5* KO MEFs did not change after the addition of BSA (Figure 5C). Finally, the decrease in phospho-S6 and phospho-S6K levels after BSA treatment in ULK1/2 DKO MEFs expressing KRasV12 was compatible levels to that of the WT MEFs harboring KRas mutant (Supplemental Figure 6). This indicates that the ability of BSA treatment to restore mTORC1 activity depends on which molecular autophagy machineries are actively involved.

### Inhibition of either autophagy or macropinocytosis sensitizes oncogenic Ras-driven PDA in combinatorial treatment with an mTOR inhibitor

**Figure 6:**
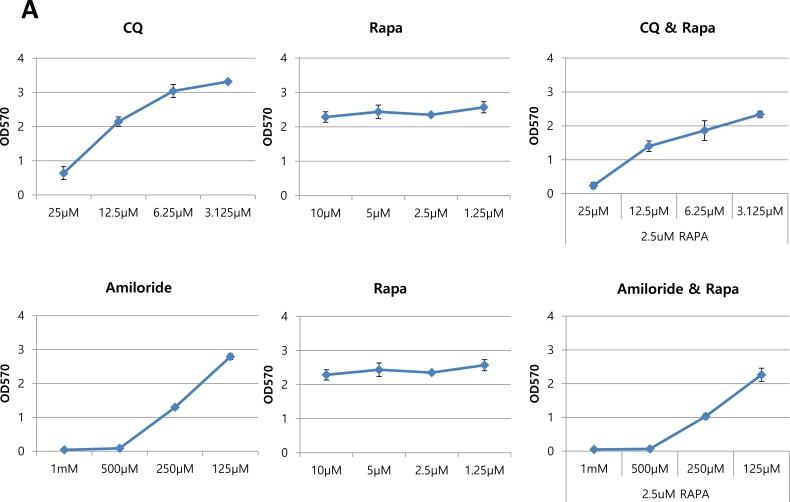

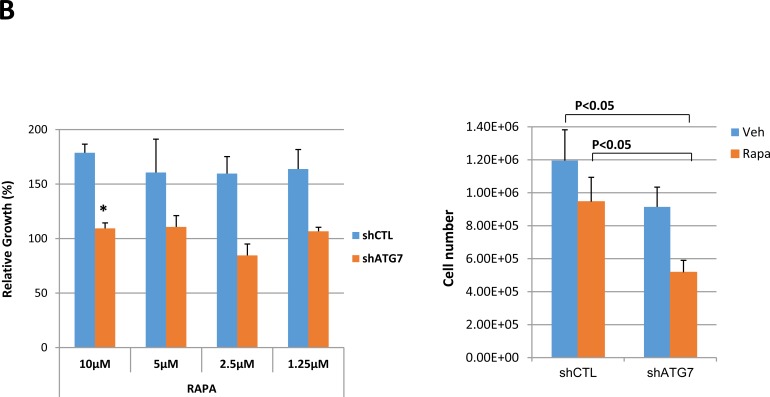

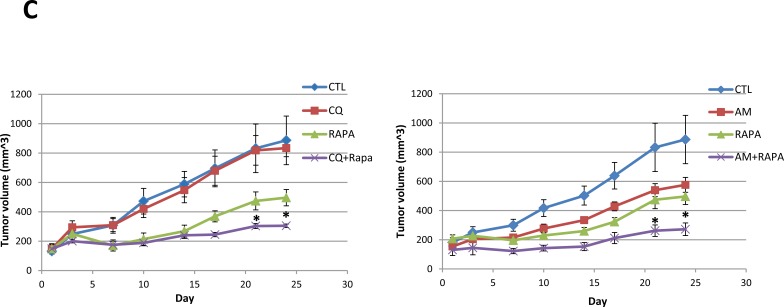
Autophagy and macropinocytosis inhibition sensitize oncogenic Ras cells in combinatorial treatment with mTOR inhibitor **A.** MIA PaCa-2 (KRas mutant) cells were treated with the indicated concentrations of CQ, amiloride, or rapamycin alone, or two of each in combination, for 72 hours. Cellular growth was assessed using an MTT assay. **B.** MIA PaCa-2 cells expressing Atg7 shRNA and control shRNA were treated with the indicated concentrations of rapamycin for 48 h and cell growth was assessed using an MTT assay. Cell growth with 5 μM rapamycin treatment was monitored by cell counting in MIA PaCa-2 expressing both Atg7 shRNA and control shRNA. Relative growth is presented after normalization by number of cells under the each condition without rapamycin treatment. **C.** Concomitant treatment with rapamycin and CQ or rapamycin and amiloride resulted in the inhibition of tumor growth in a xenograft mouse model. Subcutaneous MIA PaCa-2-driven tumors were established in 6-week old male mice. Rapamycin (1 mg kg^−1^ per day), CQ (20 mg kg^−1^ per day), or amiloride (10 mg kg^−1^ per day) alone, or two of each in combination, were administered daily via intraperitoneal injection. Tumor growth was assessed once tumor volume reached 150 mm^3^. Data are shown as the mean of five mice in each group +/−SEM. *p < 0.05. Statistical significance was determined via a student's *t*-test.

Since inhibition of mTOR increased both macropinocytosis and autophagy, we examined whether the inhibition of autophagy or macropinocytosis in combination with mTOR inhibitors suppresses cell proliferation. The growth rates of oncogenic Ras-expressing cells were assessed by an MTT assay. CQ, amiloride, and rapamycin were used as inhibitors of autophagy, a macropinocytosis and mTORC1, respectively. For combinatorial treatment, the cells were incubated with two inhibitors simultaneously for 48 hours. Growth rates of oncogenic Ras mutant cells decreased in a dose-dependent manner when treated with either the autophagy or the macropinocytosis inhibitor; treatment with rapamycin alone did not reduce cell growth *in vitro*. Simultaneous treatment with either CQ and rapamycin or amiloride and rapamycin reduced growth slightly more in MIA PaCa-2 cells compared to the treatment with any of the inhibitors alone (Figure 6A).

We also blocked autophagy genetically by stably expressing Atg7 shRNA in MIA PaCa-2 cells, and then tested cell growth following treatment with rapamycin for combinatorial effects. Cells with Atg7 knock-down showed severe growth rate reduction after rapamycin treatment compared to cells expressing control shRNA in nutrient complete conditions (Figure 6B).

Next, we examined whether this synergistic effect of combinatorial treatment can be observed in an *in vivo* model. A mouse xenograft model of MIA PaCa-2 was used to assess tumor regression with combinatorial treatment. CQ, amiloride or rapamycin were administered to the animals daily via intraperitoneal injection, as a single or combination treatment with indicated doses. Treatment with either rapamycin or amiloride alone inhibited the growth of xenografted MIA PaCa-2 tumors; CQ alone did not affect tumor growth. Combined treatment with either CQ and rapamycin or amiloride and rapamycin further inhibited MIA PaCa-2-derived tumor growth (Figure 6C).

Tumor histological phenotypes following the different drug treatments were examined using immunohistochemistry. mTORC1 activity was visualized by staining for phospho-S6 protein. Although the intensity of phsopho-S6 staining was not significantly different between single and combined treatments, levels of phospho-S6 were substantially reduced in tumors from the rapamycin-treated group (Supplemental Figure 7).

## DISCUSSION

Oncogenic Ras mutations greatly alter glucose and glutamine metabolism, supporting the proliferation and survival of cancer cells. Oncogenic Ras activation also stimulates a variety of catabolic processes, including macropinocytosis and autophagy, despite its well-known role as an anabolic signaling molecule that promotes macromolecule synthesis[12]. Here, the inhibition of BSA uptake by EIPA and the colocalization of BSA with a dextran molecular marker provide further evidence for increased macropinocytosis in cells expressing mutant Ras.

Uptake of extracellular fluid via macropinocytosis is an important way by which oncogenic Ras-expressing cells acquire the nutrients necessary to support cell growth and survival [3]. It was previously observed that oncogenic Ras-expressing cells are highly sensitivity to nutrient deprivation [4]. However, the elevated internalization of BSA observed in Ras-expressing cells may sustain cell growth despite metabolic stress induced by glutamine starvation. Consistent with that idea, PDA cells harboring oncogenic KRas generate glutamine and essential amino acids via macropinocytic degradation of extracellular proteins [3, 13]. Other cells expressing oncogenic Ras mutants display similar phenotypes when scavenging extracellular lipids [14] or internalizing extracellular ATP [15]. These catabolic phenotypes found in oncogenic Ras-mediated cancer may represent a novel resistance mechanism against metabolic stress, which helps support aggressive cell growth and survival.

Degradation of macromolecules via autophagy is also important for cell growth, as it generates molecules used for energy and macromolecule synthesis during periods of metabolic stress. Multiple growth-signaling pathways, such as the PI3K/mTOR pathway, typically suppress autophagy, whereas the activation of molecules sensitive to energy depletion, such as AMPK, facilitates autophagy [5]. Although treatment with Bafilomycin A1, a lysosomal fusion inhibitor, blocked oncogenic Ras-mediated macropinocytosis in an earlier report [3], it is not yet clear whether autophagy plays a direct role in the degradation of extracellular macromolecules. To investigate whether degradation of extracellular albumin or dextran taken up via macropinocytosis could be regulated by autophagy, cells with a subset of autophagy related genes knocked out were utilized. The accumulation of internalized macromolecules in vesicles when autophagy was inhibited indicates that autophagy could play a crucial role in the degradation of BSA and other proteins taken up during macropinocytosis. However, this role appears to be limited to Atg5- and Atg7-dependent autophagy processes; deficits in ULK-dependent autophagy did not affect macromolecule degradation.

Although autophagy is known to support tumorigenic anabolic metabolism, the regulatory mechanisms that might affect both processes are poorly understood. In this study, we found that macropinocytosis induced by oncogenic Ras activity is closely associated with autophagy, which facilitates the degradation of macropinocytic cargoes. However, not all autophagy genes have roles in this degradation process. Although double knockout of ULK1/2 is lethal in perinatal mice, similar to *atg5* or *atg7* knockout, it does not appear to be important in autophagy related to oncogenic Ras activity.

Atg5, but not ULK1/2, is part of a complex essential for elongating the autophagosomal membrane to surround the cargo complex ATG12-Atg5-Atg16L. Specifically, the E1-like enzyme Atg7 and the E2-like enzyme Atg10 conjugate Atg5 to Atg12. The Atg12-Atg5 conjugate then binds to Atg16L to from a complex with E3-like enzyme activity. Atg 7 and Atg 3 also contribute to autophagy by mediating the conjugation and localization of LC3 to lipid phosphatidylethanolamine (PE) at the autophagosomal membrane. The Atg12-Atg5-Atg16L1 complex is also required for formation of the covalent bond between LC3 and PE. Deficiencies of either Atg5 or Atg7 block the conversion of LC3 to its lipid bound form (LC3-II) [6, 16]

On the other hand, ULK, a yeast Atg1 homolog, plays a crucial role in starvation-induced autophagy and is a key downstream target of nutrient signaling molecules like mTOR and AMPK. Although the absence of ULK results in various defective molecular phenotypes, LC3-II formation occurs normally in the absence of ULK1, ULK2, and other proteins that interact with ULK, suggesting that ULK complex involved in autophagy initiation is dispensable for activating LC3 conjugating systems, which is most critical to promote the autophagy process [17]. In this report, breakdown of macropinocytic vesicles occurred normally in ULK1/2 knockout cells, but was impaired in Atg5 and Atg7 knockouts (Figure 3A; Supplemental Figure 3). Our findings support the conclusion that, while ULK is important for autophagy triggered specifically by nutrient deficiency, the roles of Atg5 and Atg7 in autophagosome membrane expansion make them crucial for autophagy more generally.

Macropinocytosis and autophagy effectively fulfill cellular metabolic demands of oncogenic Ras-mediated cancer cells. Moreover, metabolites obtained from macromolecule degradation, including amino acids, can reactivate mTORC1 and act as a feedback mechanism (Figure 5). The addition of BSA to nutrient-deficient media restored mTORC1 signaling in wild-type and ULK1/2 knockout cells, but *atg5* knockout abolished this effect (Figure 5C; Supplemental Figure 6).

Lysosomes are crucial for autophagy and are affected by mTORC1 signaling, which aids in amino acid generation via lysosomal degradation. The subcellular localization of mTOR depends on intracellular nutrient status, particularly on amino acid levels [11]. In its active state, mTOR is located at the lysosomal membrane, where it can directly access products of lysosomal degradation. In contrast, during amino acid withdrawal, inactive mTOR is no longer recruited to the lysosomal membrane but is diffusely distributed throughout the cytoplasm instead [18-23]. We show that the presence of extracellular BSA alone can relocate mTOR to lysosomal membranes in manner similar to the presence of amino acids (Figure 5B). This intracellular re-localization of mTOR by treatment with extracellular protein highlights the importance of macropinocytosis and autophagic degradation for cancer growth and survival. Furthermore, the detrimental effects of Atg5 knockout on RAS-expressing cells under amino acid deprivation suggest that cancers harboring oncogenic Ras mutations are especially susceptible to the inhibition of autophagy and/or macropinocytosis (Data not shown).

Surprisingly, we show that inactivation of mTORC1 both pharmacologically (Rapamycin) and by reducing expression (Raptor shRNA) induces macropinocytosis and enhances cancer growth (Figure 4; Supplemental Figure 5). The homeostatic balance between catabolism and anabolism, which is regulated by mTORC1, is a critical regulatory mechanism affecting cancer progression. Accordingly, the concomitant intervention of catabolism and anabolism by the treatment with CQ and rapamycin can be expected to provide the most significant anti-tumorigenic effect in mouse xenograft experiments using PDA cell line despite the weak combinatorial effect of drugs *in vitro* cell systems.

Recently, the Thompson laboratory has reported that Ras-induced macropinocytosis of extracellular proteins generates essential amino acids that activate the mTOR signaling pathway. Upregulated mTOR activity ultimately suppresses the utilization of degraded extracellular proteins as essential amino acids source [24]. Here, however, we focus on the association between autophagy and macropinocytosis. *Atg5* KO MEFs show severe impairments in extracellular protein degradation, resulting in reduced cell growth and survival under nutrient-deprived conditions. Moreover, the reduction in mTORC1 activity following treatment with extracellular BSA was not observed in *atg5* KO MEFs, demonstrating the importance of a subset of autophagy-related molecules for generating nutrients in cells with mutant Ras activity. In addition, we show that inhibiting mTORC1 activity, either pharmacologically or by reducing expression, enhance macropinocytic uptake and sustains cell growth during nutrient deprivation. Thus, catabolic and anabolic pathways are closely regulated to overcome environmental metabolic stresses in cells that express mutant Ras.

In summary, we propose a novel mechanism by which the catabolic pathways are interconnected to anabolic metabolism for facilitating tumor progression. Specifically, molecules produced by degrading extracellular proteins via macropinocytosis and autophagy, re-activates mTORC1. Furthermore, mTORC1 inhibition unexpectedly enhances these catabolic pathways, which helps provide nutrients that support cell growth and survival. Therefore, targeting autophagy and/or macropinocytosis may be an effective option for combinatorial therapy with mTOR inhibitors in oncogenic Ras-driven tumors.

## MATERIALS AND METHODS

### Chemicals and reagents

Primary antibodies against phospho-S6 (S235/236), S6 ribosomal protein, phospho-p70S6K (T389), p70S6K, LC3B, respectively were purchased from Cell Signaling Technology (Beverly, MA, USA). Primary antibodies were purchased against LAMP2 from Abcam (Cambridge, UK), P62 from Progen (Heidelberg, Germany), β-actin from Sigma-Aldrich and tubulin from Santa Cruz (Dallas, Texas, USA). The secondary antibodies, Alexa Fluor-488 conjugated anti-rabbit (#A21206) and Alexa Fluor-592 anti-mouse (#A11005), were from Life Technologies (Carlsbad, CA, USA). Horseradish peroxidase (HRP)-linked anti-rabbit and anti-mouse antibodies were from Bethyl Laboratories (Montgomery, TX, USA). Non-essential amino acids (1567906), essential amino acids (1542383), glutamine (25030081), HEPES (1627660), vitamin solution (1567870), and sodium bicarbonate (1546264) were from Life Technologies (Carlsbad, CA, USA). DAPI, fluorescein-conjugated albumin from bovine serum (BSA: A23015), tetramethylrhodamine (TMR)-conjugated albumin from bovine serum (BSA; A23016), Dextran, fluorescein (10 KDa; D1820), and Dextran, tetramethylrhodamine (10 K Da; D1817) were from Life Technologies (Carlsbad, CA, USA). Mounting media (S3023) was from DAKO (Carpinteria, CA, USA).

Bovine serum albumin (BSA; A1470), Chloroquine (CQ; C6628), Amiloride (A7410), 5-(N-Ethyl-N-isopropyl)Amiloride (EIPA; A3085), and phosphatase inhibitor cocktail were from Sigma-Aldrich. Protease inhibitor cocktail tablet was from Roche Applied Bioscience. Rapamycin (S1039) and Torin2 were purchased from Selleck Chemicals (Houston, TX, USA).

### Cell lines and culture conditions

The BxPC-3and MIA PaCa-2, human pancreatic cancer cell lines from the American Type Culture Collection (ATCC; Manassas, VA, USA) were kindly provided by YH Kim (National Cancer Center, Korea). *Ulk1/2* DKO MEFs were obtained as previously described [25]. *Atg5* KO MEFs were generated by mating *Atg5* heterozygous mice generously provided by Dr. Noboru Mizushima (Tokyo Medical and Dental University, Bunkyo-Ku, Japan) [26].

All cells were maintained under 5% CO_2_ at 37°C in medium supplemented with 10% FBS (Hyclone), 100 U/mL penicillin, and 100 μg/mL streptomycin (Life Technologies). MEFs and MIA PaCa-2 were maintained in Dulbecco's modified Eagle's medium (DMEM; Life technologies), BxPC3 were cultured in RPMI1640 (Life technologies). For glutamine starvation, DMEM without glutamine (Life Technologies) was supplemented with 10% dialyzed-FBS (Life technologies). For the amino acid starvation medium, Hank's balanced saline solution (HBSS) was supplemented with 10% dialyzed FBS, glucose, vitamins, HEPES and minerals at the same concentration as in DMEM.

Constitutively activated Ras GTPase plasmids were from Addgene. pBabe H-Ras G12V (#9051), pBabe K-Ras G12V (#9052) were generously provided from William Hahn through Addgene. MSCV HRas V12 IRES GFP (#18780) was provided from Scott Lowe through Addgene. K-Ras G12V was subcloned into pMigRI. Oncogenic Ras-expressing MEFs were generated following standard protocols for retrovirus transduction.

### Western blotting

For western blotting, cells were rinsed with ice-cold PBS and harvested using ice-cold lysis buffer (RIPA buffer). Samples were then incubated in RIPA buffer (50 mM TRIS-Cl pH 7.4, 150 mM NaCl, 1% NP-40, 0.5% Na-deoxycholate, 0.1% SDS, 1 mM EDTA) with protease inhibitor cocktail (Roche Applied Bioscience) and phosphatase inhibitor (Sigma-Aldrich) for 15 min, and soluble lysate fractions were isolated by centrifugation at 16,000 g for 15 min. Protein concentrations were determined with the Pierce BCA Protein Assay (Thermo Scientific), and equal amounts of protein were analyzed by SDS gel electrophoresis and Western blotting following standard protocols.

To measure the recovery of mTOR activity using Western blotting, the pancreatic cancer cells (BxPC3, MIA PaCa-2) were seeded and cultured in complete media for 18 h prior to amino acid deprivation. Cells were cultured in amino acid starvation medium for 1 h, and then in medium supplemented with either amino acids or 2% BSA for indicated periods of time.

### Fluorescence microscopy

For imaging of macropinocytosis, cells were incubated with 0.5 mg/ml dextran or BSA conjugated with FITC or TMR for indicated periods of time at 37°C. Subsequently, cells were washed 3 times with ice-cold PBS and fixed with 3.7% formaldehyde in PBS for 15 min. Then, nuclei were stained using DAPI and mounted using mounting media (DAKO). Images were captured using an LSM510 or LSM780 fluorescent microscope (Zeiss) and quantified using the Image J (National Institutes of Health). The total macropinosome area per cell was normalized by the area of DAPI stained nucleus of that cell; macropinosome areas were quantified in at least five distinct fields captured from different regions for an individual experimental set.

To determine the localization of mTOR, immunostaining against mTOR and LAMP2 was performed using MIA PaCa-2. After they were plated onto glass coverslips in complete medium for 18h, cells were incubated in amino acid starvation medium for 2 h and subsequently placed in medium supplemented with either amino acids or 2% BSA for indicated periods of time.

For immunostaining, cells were rinsed with ice-cold PBS, fixed with 3.7 % formaldehyde for 30 min, and permeabilized with 0.05 % Triton X-100 in PBS (PBS-T) for 5 min. After rinsing with PBS-T, cells were blocked with blocking buffer (0.5% BSA in PBS-T) for 1 h, incubated with primary antibodies in blocking buffer for 16 h in 4°C, washed three times with PBS-T and then incubated with secondary antibodies in PBS-T for 1h in room temperature. After three PBS-T washes, cells were stained with DAPI for 10 min and mounted on microscope slides. A confocal fluorescence microscope (Zeiss LSM 780) was used for imaging and analysis. Pearson's correlation coefficient was used for calculating co-localization.

### Cell growth and viability

Cells were plated in complete media on either 96 well or 24 well plates prior to starvation. Twenty-four hours after seeding, cells were washed with PBS and incubated in the indicated glutamine-or EAA starvation media with 10% dialyzed FBS. For rescue experiments, the cells were incubated in the media supplemented with 2% BSA. Negative control cells for macropinocytosis were treated with EIPA. The number of viable cells was assessed using the MTT assay and relative cell number was determined by sulforhodamine B (SRB; Sigma-Aldrich) staining following standard protocols at indicated periods of time to examine the cytotoxic effect of the drug *in vitro*. The number of cells was counted either using a Coulter Counter (Beckman Coulter) and/or using trypan blue exclusion and an automated cell counter (Nano-Entek, Korea). Cell viability was determined by Annexin V and propidium iodide (PI) staining following standard protocols at indicated periods of time (BD Biosciences). Cells negative for both Annexin V and PI staining are considered live cells.

### Mouse xenograft studies

Animal experiments were performed in accordance with protocols approved by the Institutional Animal Care and Use Committee at the National Cancer Center, Republic of Korea. Six-week-old female Balb/c nude mice (Orient Bio Inc., Korea) were handled using aseptic procedures and allowed to adjust to local conditions for 1 week before experimental manipulations began. MiaPaCa2 cells (5 × 10^6^) were mixed at a 1:1 dilution with Matrigel (BD Biosciences) and injected subcutaneously into both flanks of each mouse for a total final volume of 200 μL. When tumors reached an average volume of 150 mm^3^, rapamycin (1 mg kg^−1^ per day), CQ (20 mg kg^−1^ per day), or amiloride (10 mg kg^−1^ per day) alone or two of each in combination were administered daily via intraperitoneal injection for up to 25 days. Tumor growth was evaluated by measurement of two perpendicular diameters of the tumors, and tumor size was calculated using the formula 4π/3 × (width/2)^2^ × (length/2). The tumors were harvested and weighed at the experimental endpoint.

## SUPPLEMENTARY MATERIAL FIGURES


