# Supplementary material for: Catabolic pathways regulated by mTORC1 are pivotal for survival and growth of cancer cells expressing mutant Ras
Source: Oncotarget. 2015 Nov 14;6(38):40405–17. doi: 10.18632/oncotarget.6334 (PMC4747341; doi:10.18632/oncotarget.6334)
Supplement: Supplementary file 1 [file oncotarget-06-40405-s001.pdf]

Catabolic pathways regulated by mTORC1 are pivotal for survival and growth of cancer cells expressing mutant Ras

Supplementary Material

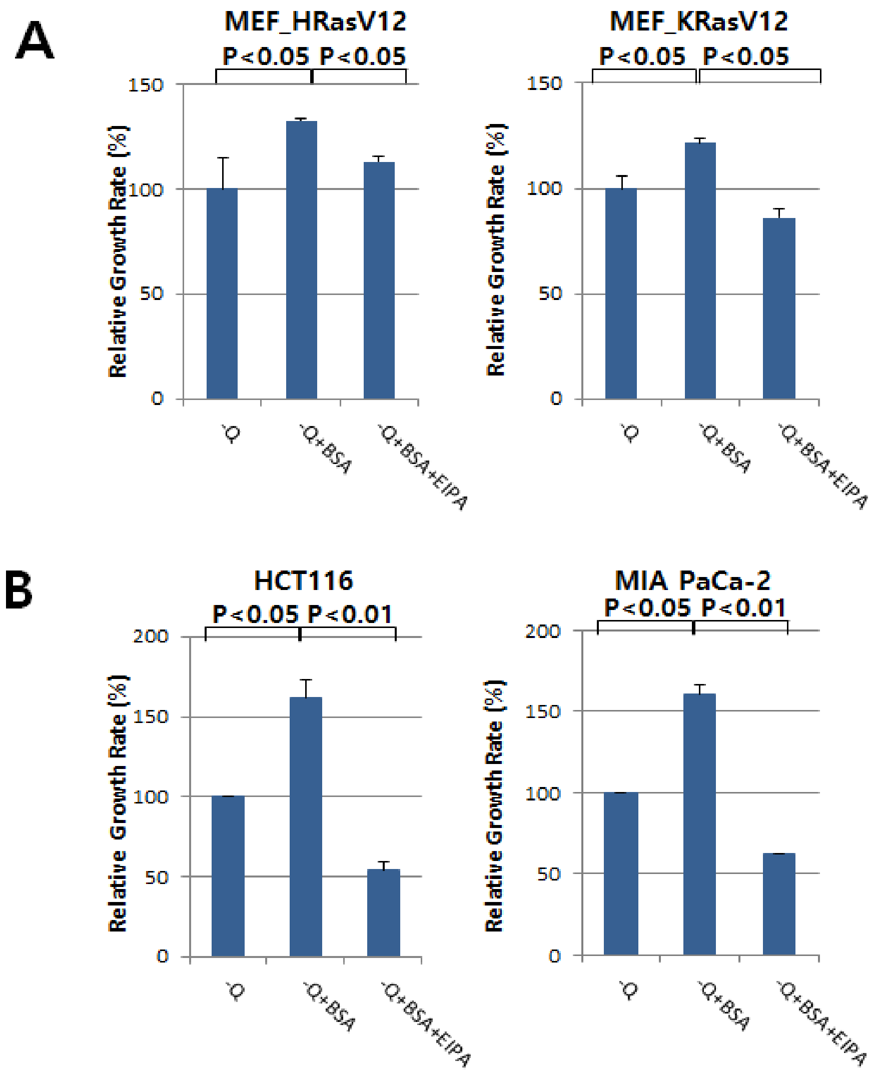

Supplemental Figure 1: When cells were cultured in glutamine-deprived conditions for 6 days with or without 2% BSA and further with EIPA treatment, cell growth rate was determined by using an MTT assay. Error bars indicate mean +/- SEM for n=3 independent experiments. Statistical significance was determined via a student's t-test.

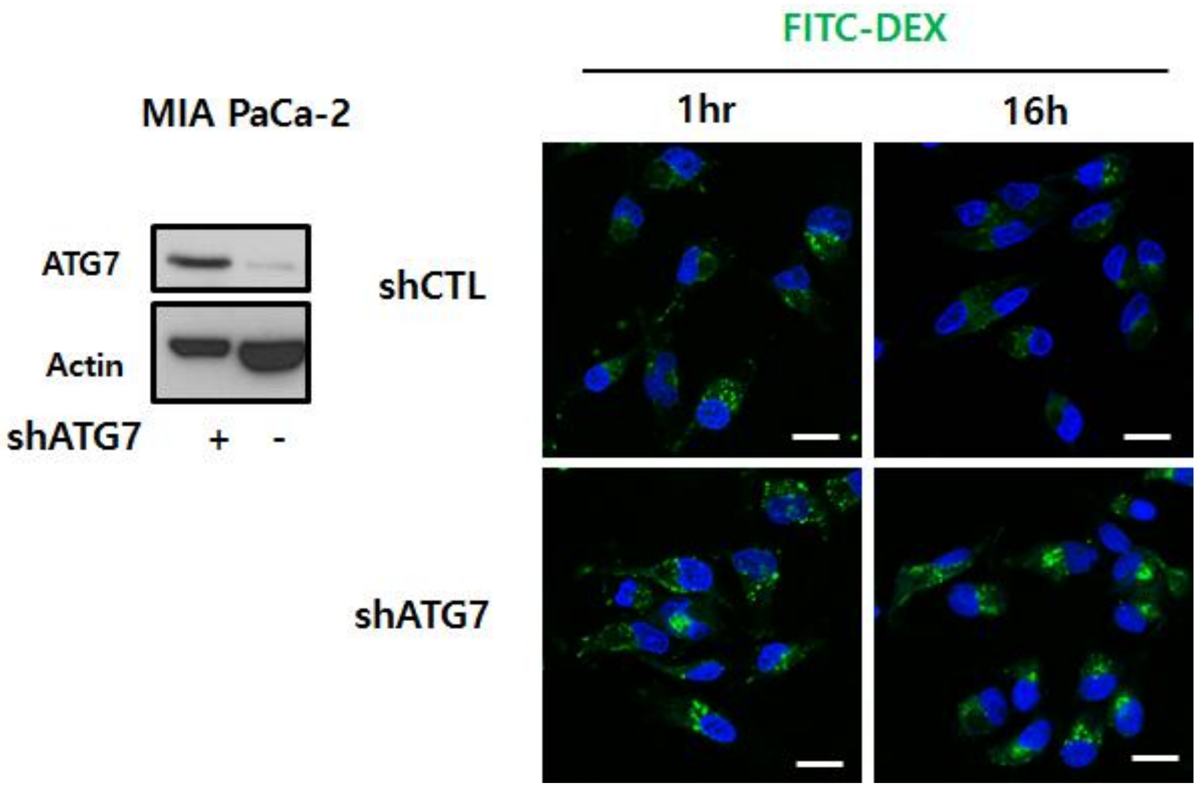

Supplemental Figure 2: Uptake of FITC-Dextran in MIA PaCa-2 expressing Atg7 shRNA and control shRNA was monitored at the indicated times. Scale bars, 20μm.

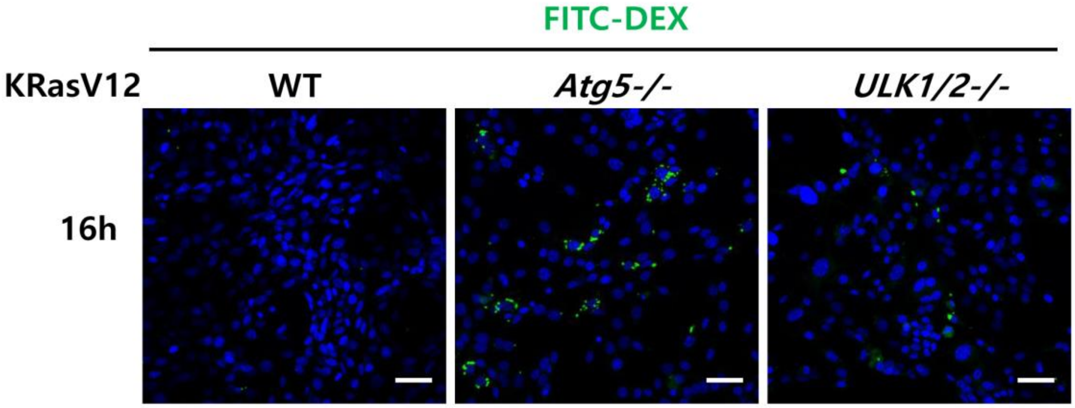

Supplemental Figure 3: Macropinocytosis in WT MEFs, *Atg5*KO(Atg5-/-) and *ULK1/2* DKO (ULK1/2-/-) MEFs expressing KRas G12V was assessed by monitoring uptake of FITC-Dextran at 16 h incubated time point. Scale bars, 50μm.

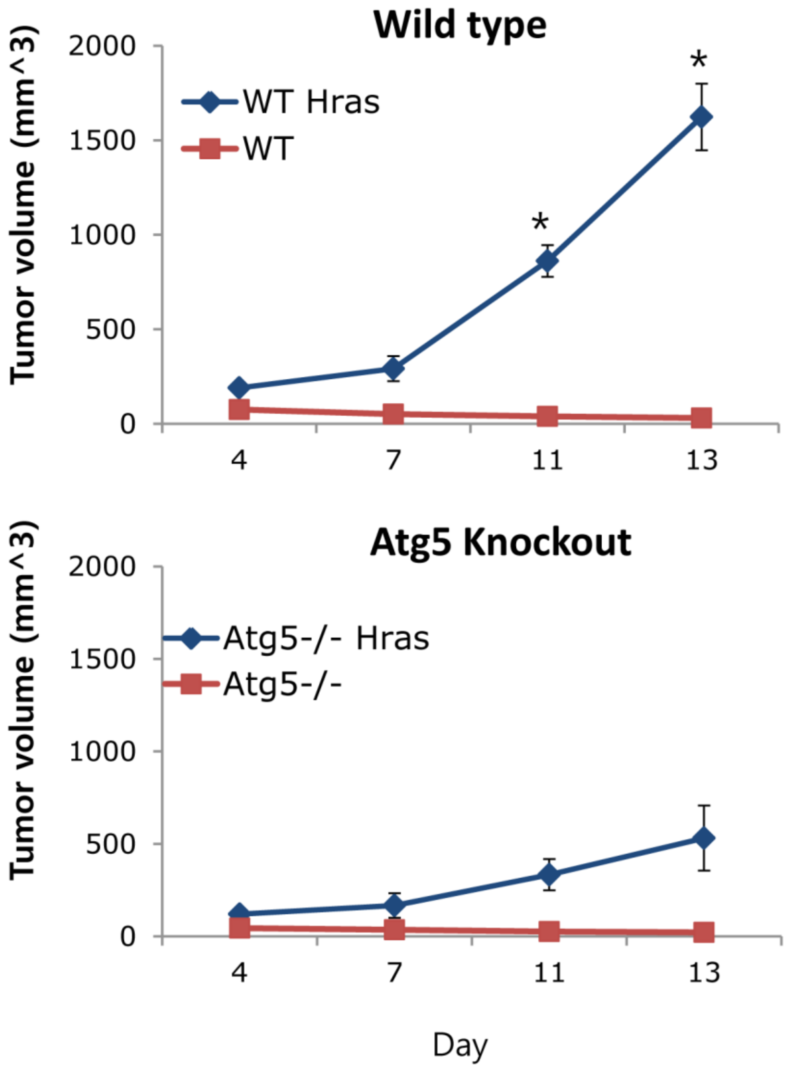

Supplemental Figure 4: HRasV12 expressing WT MEF and *Atg5*KO (Atg5-/-) cells were used for tumors establishment through subcutaneous injection in immunodeficient nude mice. Tumor size was assessed by using the formula in the Materials and Methods. Data are shown as the mean of five mice in each group +/-SEM. *p<0.05

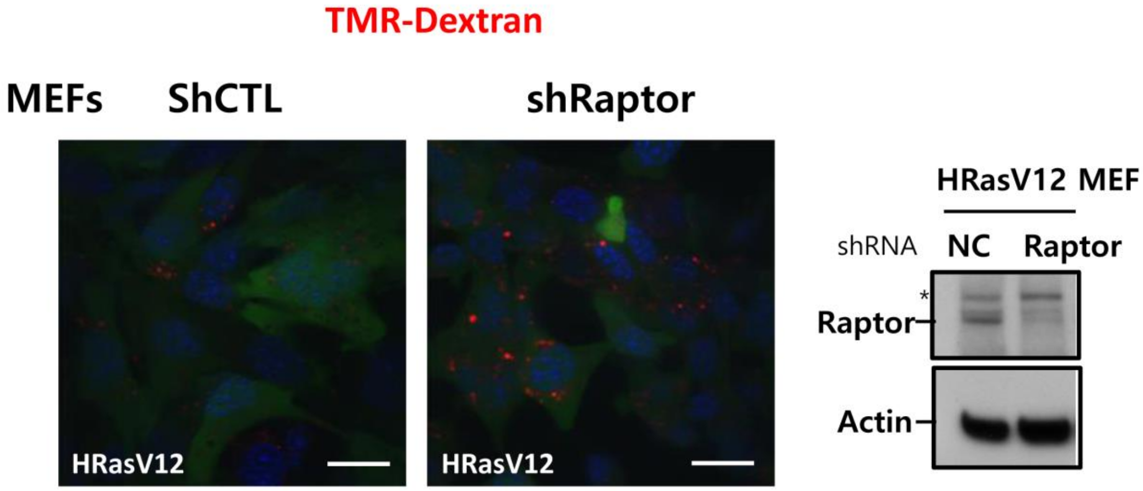

Supplemental Figure 5: Uptake of TMR-Dextran in HRasV12 MEF expressing mouse Raptor shRNA and control shRNA was monitored for macropinocytosis. Scale bars, 20 micrometer (um). Refer to supplemental figure2 legend.

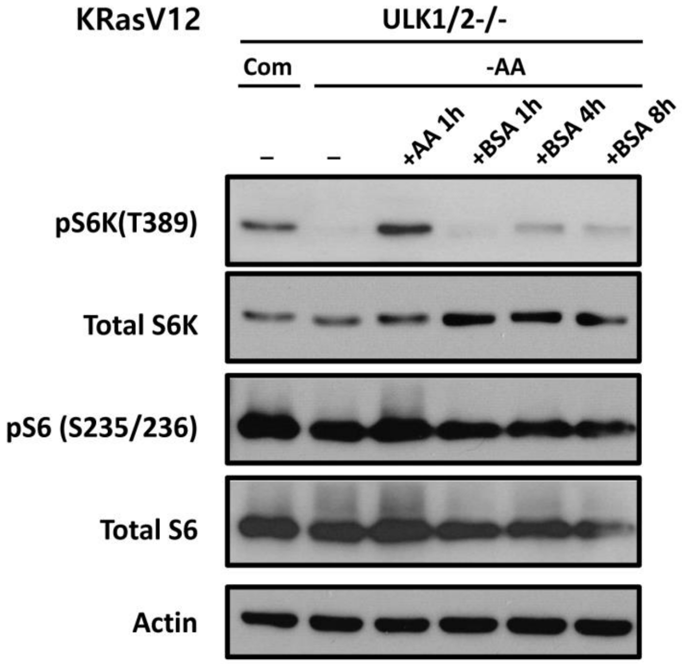

Supplemental Figure 6: KRasV12 expressing MEFs and *ULK1/2* DKO MEFs were deprived of amino acids and used for monitoring mTOR activity with 2% BSA at indicated incubation time.

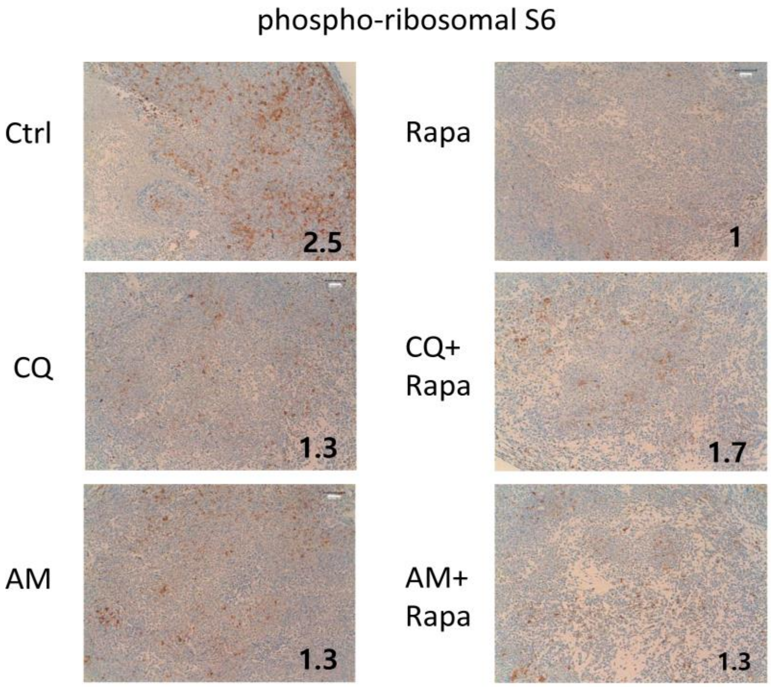

Supplemental Figure 7: Immunohistochemistry of tumor from xenograft mice in each drug. The number was used to represent intensity of protein expression.
